# mRNA and miRNA expression profile reveals the role of miR-31 overexpression in neural stem cell

**DOI:** 10.1038/s41598-020-74541-8

**Published:** 2020-10-16

**Authors:** Pengfei Li, Yuantao Gao, Xiao Li, Feng Tian, Fei Wang, Yali Wang, Bichun Zhao, Ruxin Zhang, Chunfang Wang

**Affiliations:** 1 Translational Medicine Research Center, Shanxi Medical University, Xinjian South Road 56, Taiyuan, 030001 Shanxi People’s Republic of China; 2 Nanchang University, Nanchang, 330000 People’s Republic of China; 3 Laboratory Animal Center, Shanxi Medical University, Xinjian South Road 56, Taiyuan, 030001 Shanxi People’s Republic of China

**Keywords:** Developmental biology, Neuroscience, Medical research, Molecular medicine, Neurology

## Abstract

A detailed understanding of the character and differentiation mechanism of neural stem cells (NSCs) will help us to effectively utilize their transplantation to treat spinal cord injury. In previous studies, we found that compared with motor neurons (MNs), miR-31 was significantly high-expressed in NSCs and might play an important role in the proliferation of NSCs and the differentiation into MNs. To better understand the role of miR-31, we characterized the mRNA and miRNAs expression profiles in the early stage of spinal cord-derived NSCs after miR-31 overexpression. There were 35 mRNAs and 190 miRNAs differentially expressed between the miR-31 overexpression group and the control group. Compared with the control group, both the up-regulated mRNAs and miRNAs were associated with the stemness maintenance of NSCs and inhibited their differentiation, especially to MNs, whereas the down-regulated had the opposite effect. Further analysis of the inhibition of miR-31 in NSCs showed that interfering with miR-31 could increase the expression of MNs-related genes and produce MNs-like cells. All these indicated that miR-31 is a stemness maintenance gene of NSCs and has a negative regulatory role in the differentiation of NSCs into MNs. This study deepens our understanding of the role of miR-31 in NSCs, provides an effective candidate target for effectively inducing the differentiation of NSCs into MNs, and lays a foundation for the effective application of NSCs in clinic.

## Introduction

Spinal cord injury (SCI) is the structural and functional damage of the spinal cord caused by various reasons, resulting in the impairment below the level of injury of spinal nerve function. The main characteristic of SCI is the death of cholinergic motor neurons (MNs)^1^, which is a severely disabling trauma. At the site of spinal cord injury, effective nerve regeneration rarely occurs, and injured neurons seldom repair themselves at the site of injury^2,3^. Accordingly, finding ways to improve the environment of nerve regeneration at the injured site and promote the recovery of injured MNs has become a research focus in the field of SCI treatment. After the discovery of neural stem cells (NSCs), cell transplantation has become a promising and feasible option for the treatment of SCI^4^. However, in the case of SCI model mice, almost all transplanted NSCs differentiated into glial cells^5^. In addition to the influence of the microenvironment of the injured site, the lack of understanding of the NSCs differentiation mechanism into MNs is also the main reason that the transplanted cells cannot effectively differentiate into the required neurons.

Cell differentiation is a precise process that relies on precise control over the spatial and temporal expression of transcriptional regulators, especially silencing of previously active molecules and activation of new molecular programs^6^, thus establishing clear temporal and spatial boundaries for the expression of corresponding genes^7^, ultimately triggering overall changes in cells. Studies have shown that a kind of small non-coding RNA, called microRNA (miRNA), produced by RNaseII-Dicer, can precisely regulate the expression of target genes by inhibiting the translation of mRNAs and plays an important role in various cellular processes^8,9^. Although the effect of miRNAs on single target gene inhibition is limited, each miRNA can recognize and inhibit more than hundreds of mRNA targets, and the increase or decrease of their expression may eventually lead to a comprehensive change in the gene expression profile of cells, thus providing a guarantee for the stable transformation of cell fate. The same findings have been found in the study of NSCs, such as let-7b could regulate the proliferation and differentiation of NSCs though the nuclear receptor TLX signal^10^, TLX and miR-9 could form a feedback loop to affect the differentiation of NSCs^11^, and miR-133b plays an important role in regulating maturity and functional aspects of the midbrain dopaminergic neurons^12^. Therefore, we believe that the differentiation process of NSCs can be regulated by some key miRNAs. If changing the expression of these key miRNA in the differentiation process, it may improve the differentiation proportion of NSCs to the specific neurons effectively. Based on these understandings, in order to find the miRNAs that play a key role in the differentiation of NSCs into MNs, our group compared the specific expression profiles of spinal cord-derived NSCs and MNs using TaqMan low-density array (TLDA) technology in a previous study, and analyzed the differences between them in the level of regulation of miRNAs^13^. In this study, we found that miR-31 was more than 90 times more expressed in NSCs than MNs, indicating that miR-31 mainly functions in NSCs. Studies have shown that miR-31 can promote the expansion of breast stem cells^14^ and intestinal stem cells^15^. Taken together, we can speculate that miR-31 is a key stemness maintenance gene of NSCs and may have a negative regulatory role in the differentiation of NSCs into MNs, and the study of the specific role of miR-31 in NSCs can help us further strengthen our understanding of NSCs and their differentiation mechanism into MNs.

For a certain miRNA, regardless of its interference or overexpression, the change of the initial mRNA expression profile of the cell is the most direct function of the miRNA in the cell. Therefore, in this work, we used RNA-seq analysis to determine the differential expression of genes and miRNAs after miR-31 overexpression in spinal cord-derived NSCs, and integrated them to understand the role of miR-31 in NSCs and differentiation.

## Result

### miR-31 differential expression between overexpression group and control group

**Figure 1:**
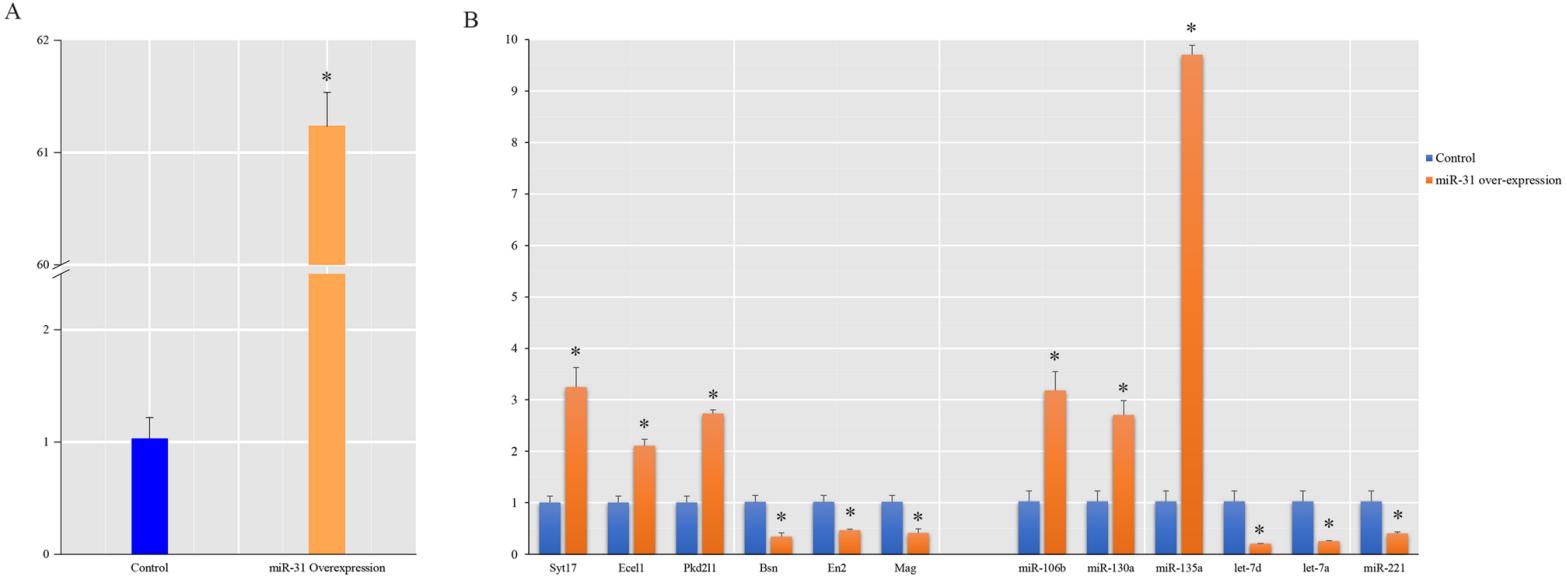
The q-PCR result of miR-31 and some DEGs, DEmiRNAs after miR-31 overexpression. (**A**) Compared with the control group, the miR-31 overexpression group had significantly higher miR-31 levels. (*Indicates p ≤ 0.05, compared with its control group.). (**B**) Experiments to verify the reliability of sequencing results showed that the q-PCR results of randomly selected DEGs and DEmiRNAs had the same trend as their sequencing results. (*Indicates p ≤ 0.05, compared with its control group).

We analyzed the miR-31 difference expression between the miR-31 overexpression group and the control group by q-PCR, and found that the expression of miR-31 in the overexpression group was 61.2 times higher than that in the control group (Fig. 1A). This means that our overexpression system can effectively improve the expression of miR-31 in NSCs.

### mRNA sequencing data mapping and annotation

A total of 2 cDNA libraries were sequenced from the miR-31 overexpression group and miR-31 overexpression control group. After removing the adaptors and filtering, 35,173,328 clean reads were obtained from the miR-31 overexpression group and 35,353,796 clean reads were obtained from control group. Then we compared the beads with the reference genome sequences by using TopHat2 software^16^. We found that 84.25% reads were successfully aligned in miR-31 overexpression group and 84.10% reads were successfully aligned in control group.

### miRNA sequencing data mapping and annotation

A total of 2 cDNA libraries were sequenced from the miR-31 overexpression group and miR-31 overexpression control group. After removing reads with low quality, trimming the 3′adapter and discarding the sequences shorter than 18 nt and longer than 30 nt, 15,266,203 clean reads were obtained from the miR-31 overexpression group and 13,923,446 clean reads were obtained from control group. Using Bowtie^17^ software, clean Reads were aligned with Silva database, GtRNAdb database, Rfam database and Repbase database respectively. Unannotated reads containing miRNAs were obtained by filtering ncRNAs such as ribosomal RNA (rRNA), transport RNA (tRNA), intranuclear small RNA (snRNA), nucleolar small RNA (snoRNA) and repetitive sequences. The known and new miRNAs were identified by using the software of miRDeep2^18^.

### DEGs and DEmiRNAs between miR-31 overexpression group and control group

There were 35 DEGs between the miR-31 overexpression group and the control group. Among these DEGs, 22 (62.9%) genes were down-regulated while 13 (37.1%) genes were up-regulated in the miR-31 overexpression group compared with control group, and 9 (25.7%) genes were the new genes (Supplementary Table S3).

After miRNA-Seq, we obtained 1063 miRNAs, and 980 miRNAs were known miRNAs, others were new predicted miRNAs. Among them, there were 190 miRNAs differentially expressed between overexpression group and its control group. 20 (10.5%) miRNAs were down-regulated while 169 (89.5%) miRNAs were up-regulated in the miR-31 overexpression group compared with control group, and 17 (8.9%) miRNAs may be the new miRNAs (Supplementary Table S4).

### The results of q-PCR validated the credibility of sequencing results

To validate the sequencing results, we investigated the relative expression levels by randomly selecting 6 mRNAs (Bsn, Ecel1, En2, Mag, PKD2L1, Syt17) and 6 miRNAs (mmu-let-7a-5p, mmu-let-7d-5p, mmu-miR-106b-5p, mmu-miR-130a-5p, mmu-miR-135a-5p, mmu-miR-221-5p) by q-PCR (Fig. 1B), and the results showed that there was same trend of difference between q-PCR results and sequencing results, which indicated the reliability of the sequencing analysis results.

### Functional analysis of DEGs

After functional annotation, the numbers of all DEGs annotated to each database are shown in Table 1.

**Table 1:** DEGs annotation result statistics.

COG	GO	KEGG	Swiss-Prot	NR	Total
12	28	16	32	34	34

**Figure 2:**
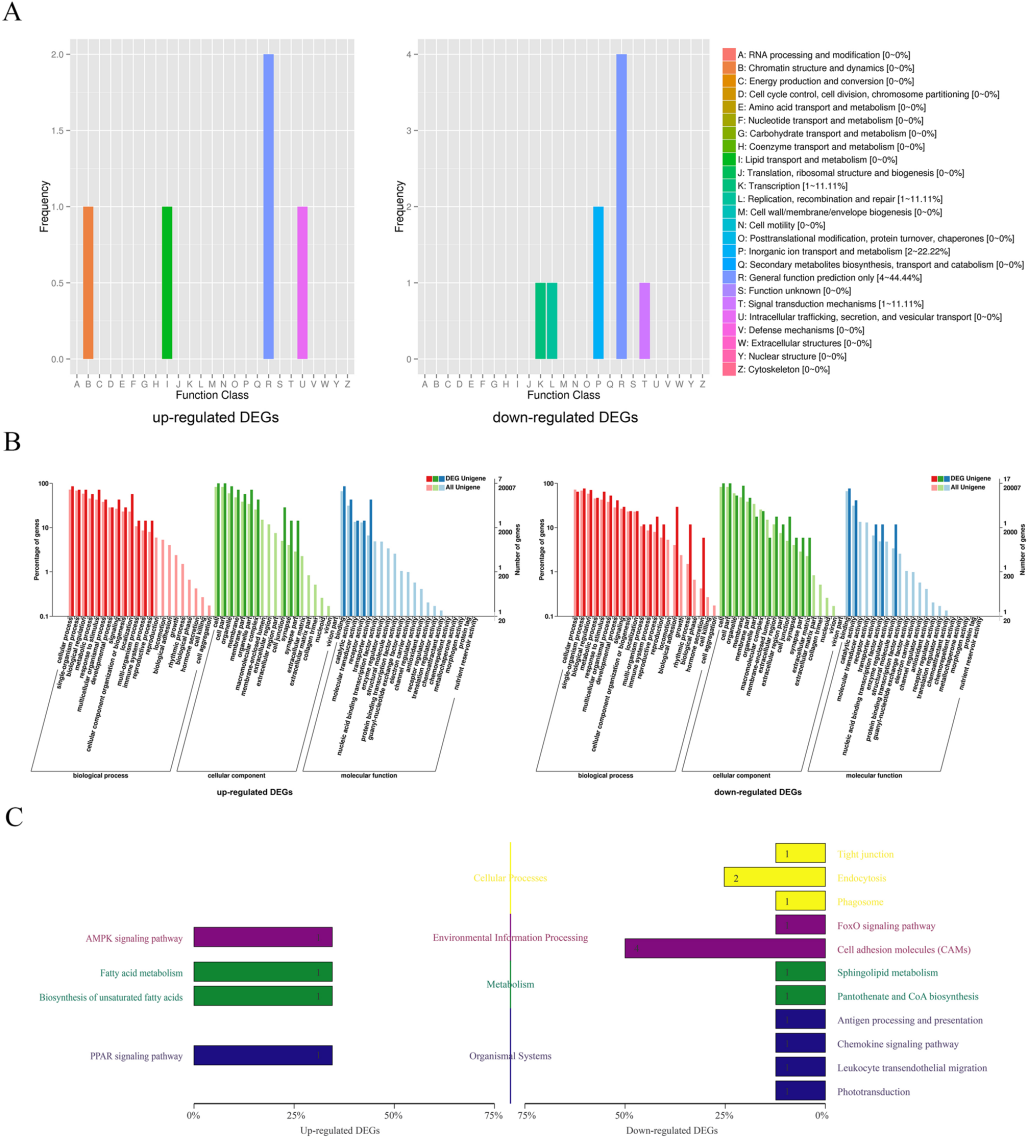
Annotated statistical chart of DEGs. (**A**) Statistical map of COG annotation classification of DEGs showed the up-regulated DEGs were mainly distributed in chromatin structure and dynamics, lipid transport and metabolism, intracellular trafficking, secretion, and vesicular transport. And the down-regulated DEGs were mainly distributed in translation, replication, recombination and repair, signal transduction mechanisms, inorganic ion transport and metabolism. (**B**) Statistical map of GO annotation classification of DEGs showed that the up-regulated DEGs were mainly distributed in the reproductive process, localization and developmental process and signaling of the BP cluster, the membrane part, cell junction, and synapse part of the CC cluster, and the transporter activity, catalytic activity of the MF cluster. The down-regulated DEGs were distributed in the hormone secretion, rhythmic process and biological adhesion of the BP cluster, the extracellular matrix, synapse part and cell junction of the CC cluster, and the structural molecule activity, nucleic acid binding transcription factor activity and transporter activity of the MF cluster. C. KEGG classification map of DEGs showed the up-regulated DEGs were mainly concentrated in the AMPK signaling pathway (Environmental information processing), and PPAR signaling pathway (Organismal systems). The down-regulated DEGs were mainly concentrated in endocytosis (Cellular processes) and cell adhesion molecules (Environmental information processing).

COG (Cluster of Orthologous Groups of proteins) database is based on the phylogenetic relationship of bacteria, algae and eukaryotes, which can be used for orthologous classification. Among different functional classes, the proportion of genes reflects the metabolic or physiological bias in the corresponding period and environment. Figure 2A showed the up-regulated DEGs were mainly distributed in chromatin structure and dynamics, lipid transport and metabolism, intracellular trafficking, secretion, and vesicular transport of the COG classification. And the down-regulated DEGs were mainly distributed in translation, replication, recombination and repair, signal transduction mechanisms, inorganic ion transport and metabolism of the COG classification.

GO annotated DEGs mainly belonged to the three functional clusters (biological process, BP; cellular component, CC; molecular function, MF). The GO annotation classification statistical graph shows the number of genes annotated to the pathway and their proportion to the total number of genes annotated, and reflects the status of the secondary functions of GO in the context of DEGs as well as all genes. The obvious proportion difference indicates that the proportion trend of DEGs and all genes under this secondary function is different, and the function may be closely related to the expression difference. Figure 2B showed that compared with the whole genetic background, the main differences in the up-regulated DEGs distribution trend were the reproductive process, localization and developmental process and signaling of the BP cluster, the membrane part, cell junction, and synapse part of the CC cluster, and the transporter activity, catalytic activity of the MF cluster. The main differences in the down-regulated DEGs distribution trend were the hormone secretion, rhythmic process and biological adhesion of the BP cluster, the extracellular matrix, synapse part and cell junction of the CC cluster, and the structural molecule activity, nucleic acid binding transcription factor activity and transporter activity of the MF cluster.

The KEGG (Kyoto Encyclopedia of Genes and Genomes) database is the main public database on metabolic pathways. The KEGG classification map shows the number of genes annotated to this pathway and their proportion to the total number of genes annotated. Figure 2C showed the up-regulated DEGs were mainly concentrated in the AMPK signaling pathway (Environmental information processing), and PPAR signaling pathway (Organismal systems). The down-regulated DEGs were mainly concentrated in endocytosis (Cellular processes) and cell adhesion molecules (Environmental information processing).

GO and KEGG enrichment analysis found that only 4 down-regulated DEGs were significantly enriched in cell adhesion molecules (CAMs) in KEGG enrichment analysis, and there were no other significant enrichments.

### Functional analysis of DEmiRNA

After functional annotation, the number of all potential target genes (PTGs) of DEmiRNA annotated to each database were shown in Table 2.

**Table 2:** Annotation result statistics.

COG	GO	KEGG	Swiss-Prot	NR	Total
2198	5966	3359	6553	6549	6553

**Figure 3:**
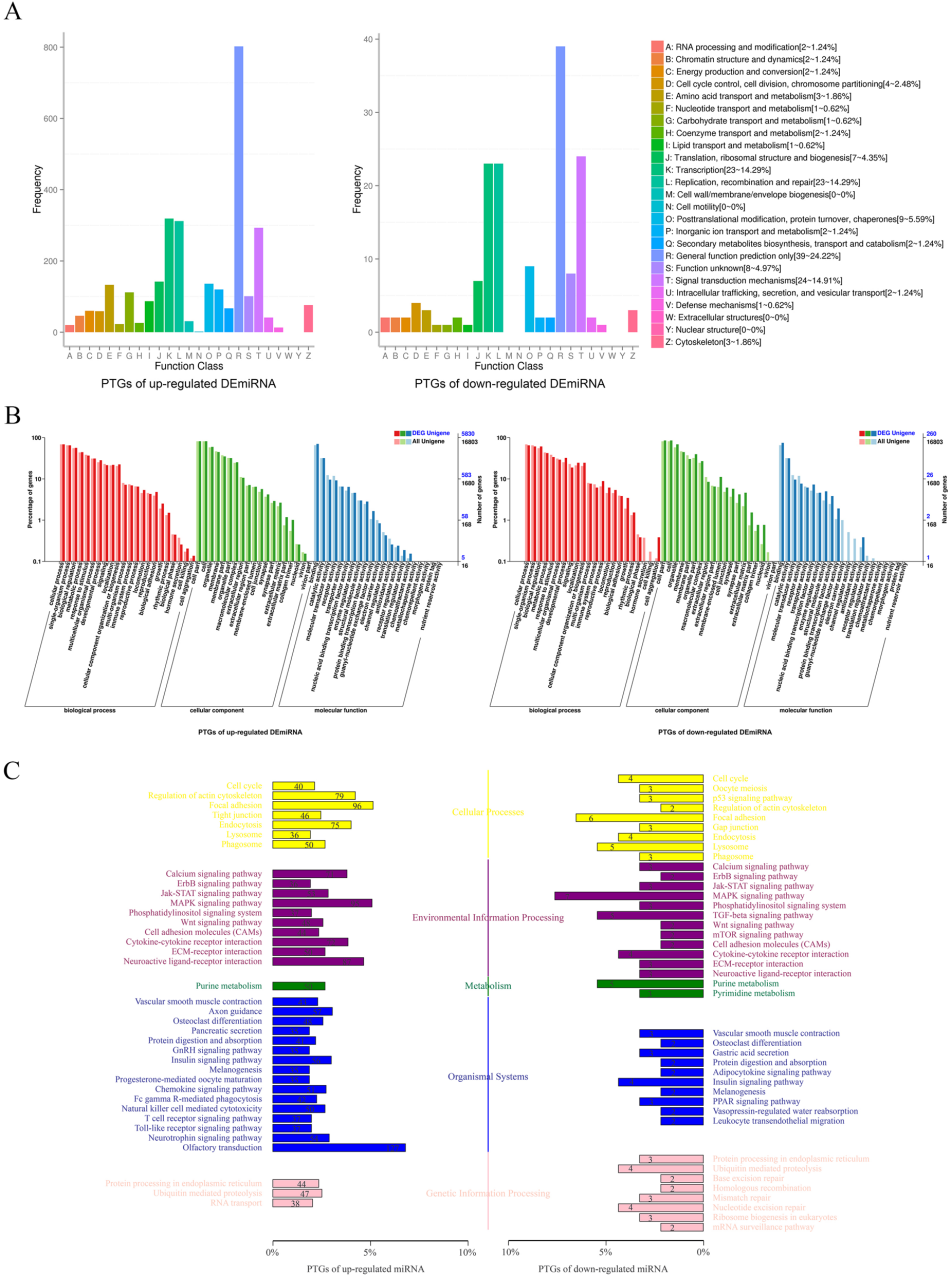
Annotated statistical chart of PTGs. (**A**) Statistical map of COG annotation classification of PTGs showed that in addition to the general function prediction only of COG classification, no matter the up or down regulated DEmiRNAs, distribution of PTGs were mostly in transcription, replication, recombination and repair, signal transduction mechanisms. (**B**) Statistical map of GO annotation classification of PTGs showed that the main differences in the up-regulated DEmiRNAs PTGs were mainly distributed in the biological adhesion, growth and rhythmic process of the BP cluster, the synapse, extracellular matrix part and collagen trimer of the CC cluster, and the nucleic acid binding transcription factor activity, guanyl-nucleotide exchange factor activity, and the protein binding transcription factor activity of the MF cluster. The down-regulated DEmiRNAs PTGs were mainly distributed in the reproductive process, growth and cell aggregation of the BP cluster, the nucleoid, extracellular matrix part and synapse part of the CC cluster, and the receptor regulator activity, guanyl-nucleotide exchange factor activity, and structural molecule activity of the MF cluster. (**C**) KEGG Classification Map of PTGs showed the PTGs of the up-regulated DEGs were mainly concentrated in the focal adhesion (Cellular processes), MAPK signaling pathway (Environmental information processing), purine metabolism (Metabolism), axon guidance (Organismal systems) and ubiquitin mediated proteolysis (Genetic information processing). The PTGs of the down-regulated DEGs were mainly concentrated in focal adhesion (Cellular processes) and MAPK signaling pathway (Environmental information processing), purine metabolism (Metabolism), insulin signaling pathway (Organismal systems) and ubiquitin mediated proteolysis (Genetic information processing).

Figure 3A showed that in addition to the general function prediction only of COG classification, no matter the up or down regulated DEmiRNAs, distribution of PTGs were mostly in transcription, replication, recombination and repair, signal transduction mechanisms.

Figure 3B showed that compared with the whole genetic background, the main differences in the distribution trend of up-regulated DEmiRNAs PTGs were the biological adhesion, growth and rhythmic process of the BP cluster, the synapse, extracellular matrix part and collagen trimer of the CC cluster, and the nucleic acid binding transcription factor activity, guanyl-nucleotide exchange factor activity, and the protein binding transcription factor activity of the MF cluster. The main differences in the distribution trend of down-regulated DEmiRNAs PTGs were the reproductive process, growth and cell aggregation of the BP cluster, the nucleoid, extracellular matrix part and synapse part of the CC cluster, and the receptor regulator activity, guanyl-nucleotide exchange factor activity, and structural molecule activity of the MF cluster.

Figure 3C showed the PTGs of the up-regulated DEGs were mainly concentrated in the focal adhesion (Cellular processes), MAPK signaling pathway (Environmental information processing), purine metabolism (Metabolism), axon guidance (Organismal systems) and ubiquitin mediated proteolysis (Genetic information processing). The PTGs of the down-regulated DEGs were mainly concentrated in focal adhesion (Cellular processes) and MAPK signaling pathway (Environmental information processing), purine metabolism (Metabolism), insulin signaling pathway (Organismal systems) and ubiquitin mediated proteolysis (Genetic information processing).

**Figure 4:**
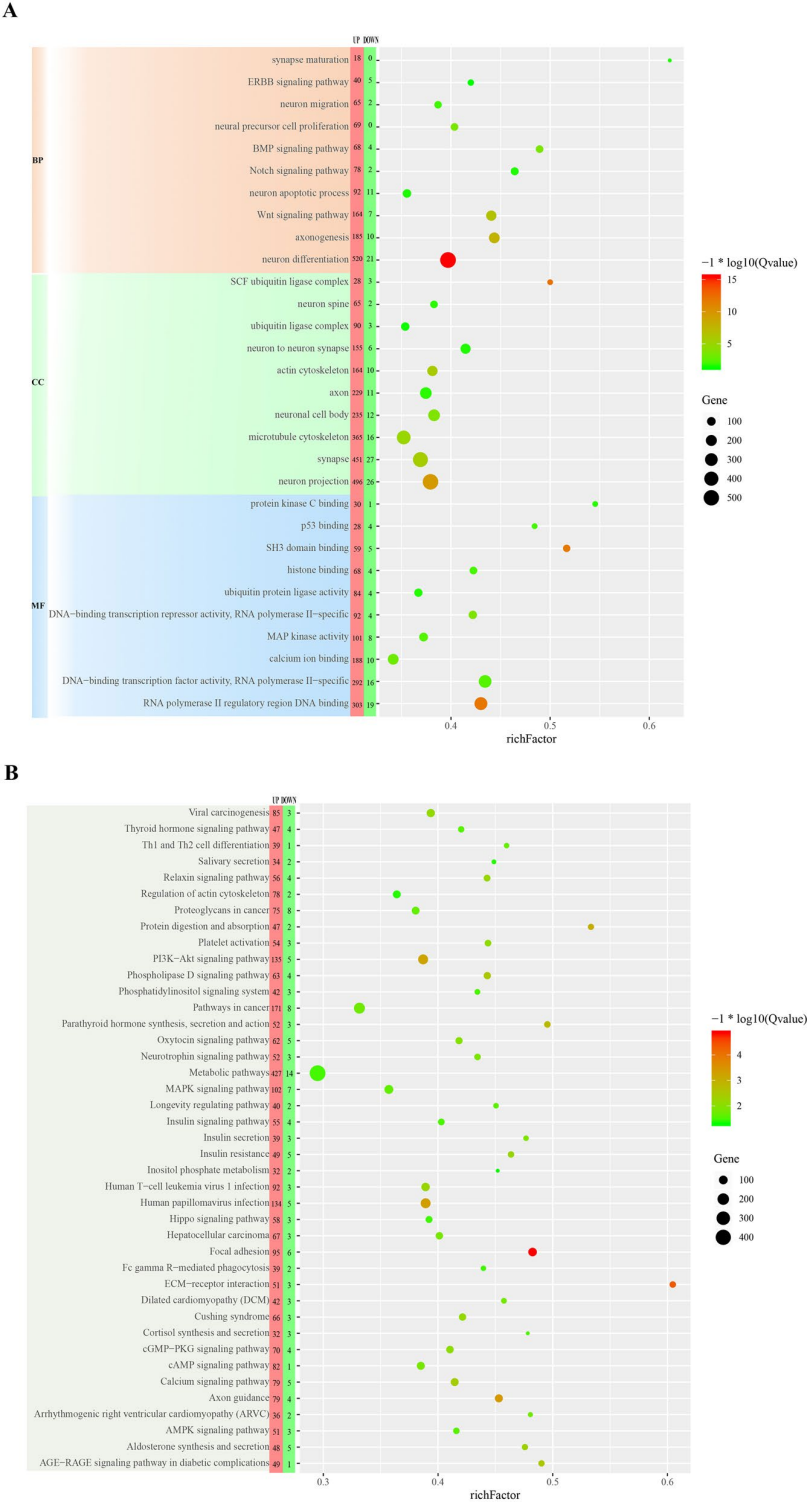
The results of GO and KEGG enrichment analysis of PTGs. (**A**) The GO categories associated with nervous system from GO enrichment analysis of PTGs showed that PTGs were mainly grouped into the neuron differentiation, axonogenesis and Wnt signaling pathway of the BP cluster, the neuron projection and synapse of the CC cluster, and RNA polymerase II regulatory region DNA binding of the MF cluster. (**B**) The results of KEGG enrichment analysis of PTGs showed the significantly enriched KEGG pathways of PTGs were metabolic pathways, focal adhesion, PI3K-Akt signaling pathway.

After enrichment analysis, we selected GO categories associated with nervous system to plot Fig. 4A, which showed that PTGs were mainly grouped into the neuron differentiation, axonogenesis and Wnt signaling pathway of the BP cluster, the neuron projection and synapse of the CC cluster, and RNA polymerase II regulatory region DNA binding of the MF cluster. Figure 4B showed the significantly enriched KEGG pathways of PTGs were metabolic pathways, focal adhesion, PI3K-Akt signaling pathway.

### The integration analysis of the DEGs and DEmiRNAs

**Figure 5:**
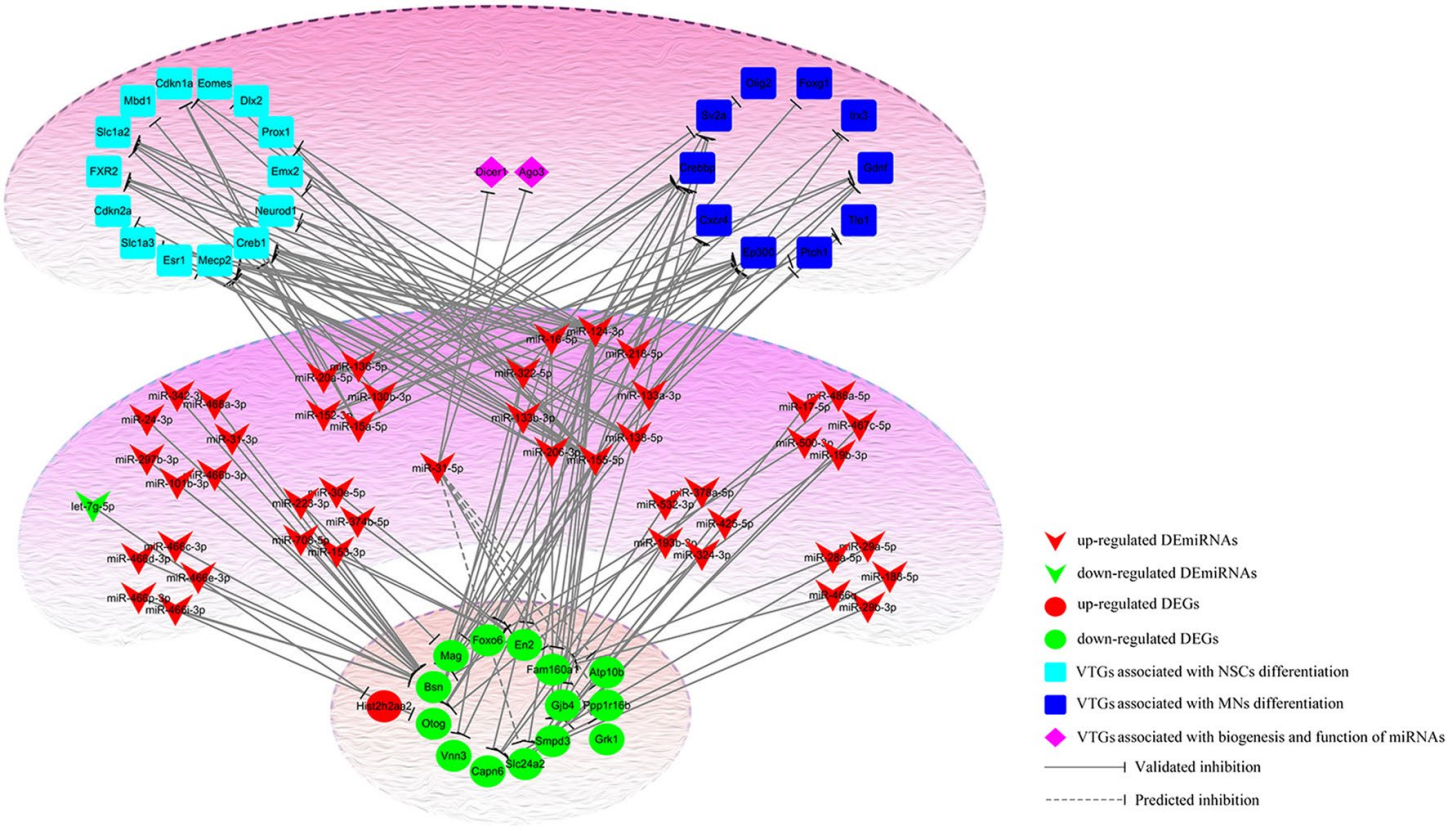
The regulatory relationship between DEmiRNAs, DEGs and VTGs showed that there were complex regulatory relationships between them. Among them, 43 DEmiRNAs and 12 DEGs had opposite expression patterns, suggesting a regulatory relationship between them. VTGs of DEmiRNAs with opposite expression patterns to DEGs revealed that about 10 VTGs were MNs differentiation-related genes and 14 VTGs were NSCs differentiation-related genes.

Using DIANA-TarBase V.8 and miRWalk, we obtained VTGs of DEmiRNAs and then cross-analyzed them with DEGs. Since the effect of miRNAs on target genes is mainly to inhibit their expression, we constructed a regulatory network of miRNA-mRNA based on the opposite expression patterns of DEGs, VTGs and DEmiRNAs (Supplementary Table S5, Fig. 5). The results showed that 43 DEmiRNAs and 12 DEGs had opposite expression patterns, suggesting a regulatory relationship between them. In addition, further analysis of PTGs and VTGs revealed that 4 DEGs (Atp10b, Grk1, Ppp1r16b and Slc24a2) were PTGs of miR-31-5p and the miRNA biogenesis gene Dicer1 and inhibition-related genes Ago3 were VTGs of miR-31-5p. Analysis of VTGs of DEmiRNAs with opposite expression patterns to DEGs revealed that about 10 VTGs were MNs differentiation-related genes and 14 VTGs were NSCs differentiation-related genes.

**Figure 6:**
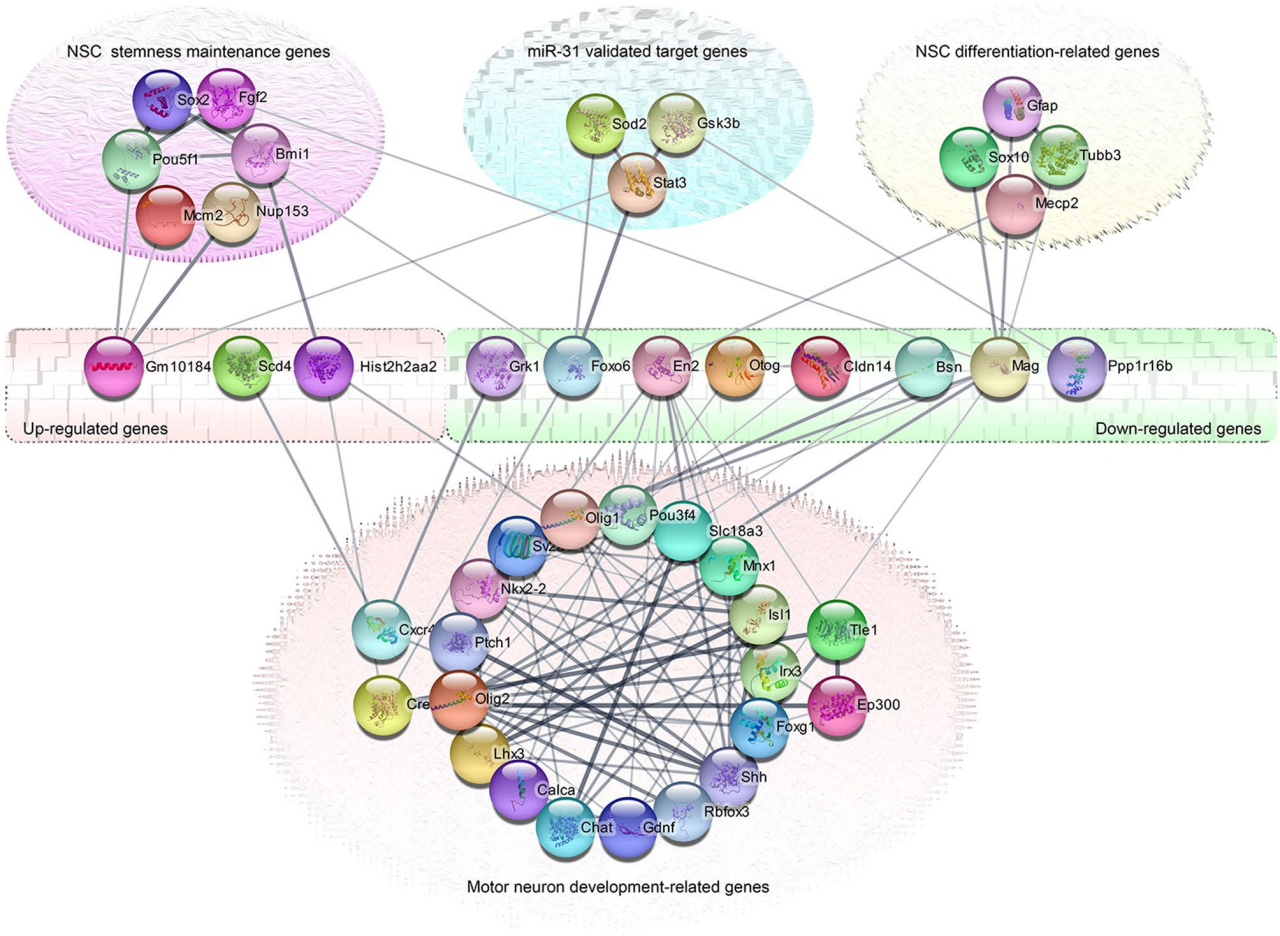
The PPI network between DEGs and VTGs showed that the DEGs could established complex interaction networks through some intermediate node genes. Most of these intermediate node genes were not only key genes related to MNs differentiation, NSCs differentiation or stemness maintenance, but also VTGs of DEmiRNAs, especially some of them were the VTGs of miR-31.

To further understand the PPI network between DEGs and VTGs, we analyzed them using String database (Fig. 6). The results showed that although DEGs had no direct interaction relationship with each other, they established complex interaction networks through some intermediate node genes. Most of these intermediate node genes are not only key genes related to MNs differentiation, NSCs differentiation or stemness maintenance, but also VTGs of DEmiRNAs, especially some of them are VTGs of miR-31.

### Expression of NSCs and MNs related genes after overexpression or interference with miR-31

**Figure 7:**
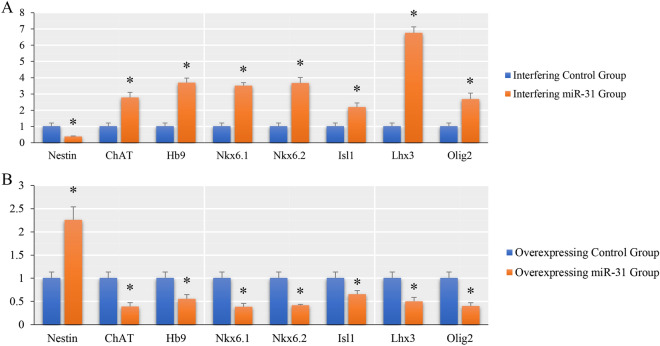
The q-PCR results of NSCs and MNs related genes after overexpression or interference with miR-31. (**A**) The q-PCR results of NSCs and MNs related genes after miR-31 overexpression (*indicates p ≤ 0.05, compared with its control group.). (**B**) The q-PCR results of NSCs and MNs related genes after inhibition of miR-31 (*indicates p ≤ 0.05, compared with its control group).

To further understand the role of miR-31 in NSCs, we investigated the expression of NSCs and MNs-related genes after overexpression or interference with miR-31. The results (Fig. 7) showed that after interference with the expression of miR-31, the expression of Nestin, a specific marker of NSCs, decreased compared with the control group, while the expression of ChAT, Hb9, Nkx6.1, Nkx6.2, Isl1, Lhx3 and Olig2, which are related to MNs, increased to different degrees. After overexpression of miR-31, the expression of Nestin increased and the expression of MNs-related genes ChAT, Hb9, Nkx6.1, Nkx6.2, Isl1, Lhx3 and Olig2 decreased compared with the control group. (There was a significant difference between the experimental group and the control group, P ≤ 0.05).

## Discussion

Since a single miRNA can act on hundreds or thousands of target genes, the alternation of its expression not only lead to changes of the corresponding target gene expression level, but also can cause cascade radiation-like changes in the cell through protein–protein interactions, just like the butterfly effect, and ultimately even fundamentally convert the cell fate. In the past few years, many miRNAs have been demonstrated to be involved in the stemness maintenance of NSCs^19^ and the differentiation of MNs^7^. But the specific details and cascade of their roles are still unclear. Our previous studies showed that compared with MNs, miR-31 was highly expressed in NSCs, and the difference was very obvious, which suggesting that miR-31 had the opposite regulatory role in NSCs and MNs. It had been confirmed that miR-31 played an important role in the mode of stem cell division^20,21^. For stem cells, symmetric cell division and asymmetric cell division are their unique renewal and differentiation mechanisms^22^. Therefore, a detailed study of the role of miR-31 in NSCs will help to understand the stemness maintenance of NSCs and the mechanism of MNs differentiation, so as to better apply NSCs to the treatment of MNs injury. In this study, we conducted some preliminary studies on the role of miR-31 in NSCs by studying the changes of mRNA and miRNA expression profiles in the early stage of miR-31 overexpression.

Although the number of genes with obvious differences in the early stage of NSCs after miR-31 overexpressing was small and there was no obvious trend of GO and KEGG pathway clustering, the distribution of these DEGs in COG, GO and KEGG showed that the up-regulated DEGs were mainly attributed to localization (GO) and metabolism (KEGG), while the down-regulated DEGs were mainly attributed to inorganic ion transport and metabolism (COG), biological adhesion (GO) and cell adhesion molecules (KEGG). These indicate that the direct effect of overexpressing miR-31 on NSCs is also associated with these GO categories and KEGG pathways. Since the role of miRNAs is mainly to inhibit the expression of target genes, down-regulated DEGs were the main objects of our analysis. Studies have shown that Bsn, namely Bassoon, was a presynaptic marker^23^; En2 gene encodes a transcription factor containing homeobox, which participated in the development of embryonic midbrain-hindbrain and could promote the differentiation of NSCs into GABAergic neurons^24^; Mag played an important role in neurite outgrowth^25^; and smpd3 was mainly expressed in neurons of the central nervous system^26^. These genes were closely related to the differentiation of NSCs, and their expression declines after miR-31 overexpression. These suggested that miR-31 overexpression could inhibit the differentiation of NSCs. PKD2L1, which belong to the up-regulated DEGs, was a transient receptor potential channel, mainly expressed in spinal cerebrospinal fluid-contacting neurons^27^. The NSCs we selected in this experiment were obtained from the embryonic spinal cord. In the spinal cord, NSCs resided in the ependymal region around the central canal^28^, which were in close contact with circulating cerebrospinal fluid, and migrated out of this region only when differentiated. The increased expression of PKD2L1 suggested that miR-31 overexpression could further maintain the location of NSCs in the spinal cord to adapt to their niche, and also hinted that miR-31 had a certain effect on the stemness maintenance of NSCs.

Our study found that the number of DEmiRNAs in early stage of NSCs after miR-31 overexpressing was much more than DEGs, which suggested that the direct role of miR-31 in NSCs was mainly achieved by changing the expression of numerous miRNAs, which in turn played a regulatory role against their own target genes, thereby expanding the regulatory effect of miR-31. GO and KEGG cluster analysis for PTGs showed that they were mainly enriched in neuron differentiation and RNA polymerase II regulatory region DNA binding (GO), metabolic pathways and PI3K-Akt signaling pathway (KEGG). These GO categories and KEGG pathways are closely related to the proliferation and differentiation of NSCs. Studies have shown that miR-106b could promote the renewal of NSCs and inhibit their differentiation^29^; miR-130a and miR-138 could inhibit axon growth and regeneration^30,31^; miR-20b overexpression could downregulate the expressions of Map2 and Tubb3 (well-known neuronal markers)^32^. In this study, we observed that these miRNAs were upregulated in the early stage of NSCs after miR-31 overexpression. It has also been confirmed that let-7a was mainly involved in neuronal differentiation^33^; let-7d could reduce the proliferation of NSCs and promote neuronal differentiation and migration when overexpressed in vivo^34^; and overexpression of miR-221 could induce neuronal differentiation of PC12 cells^35^. These miRNAs were all among the down-regulated DEmiRNAs in our study. Taken together, these findings suggest that in the early stage of NSCs after miR-31 overexpression, the expression of miRNA that can promote its proliferation and renewal and inhibit differentiation is up-regulated in NSCs, while the expression of miRNA that have the opposite effect is down-regulated, further suggesting that the main role of miR-31 on NSCs is to maintain stemness and inhibit their differentiation.

Through the study of interaction analysis between DEGs, DEmiRNAs and their VTGs and PTGs, we observed that there were complex regulatory and interaction networks between them. Among these interacting genes, a large number of DEmiRNAs showed negative regulatory effect on more than half of the DEGs. The up-regulated DEGs were mainly related to the stemness maintenance genes of NSCs, among them, Sox2 and Nup153 could maintain NSCs stemness^36,37^; Bmi-1 and Pou5f1 played a role in NSCs self-renewal and proliferation^38,39^; Mcm2 was one of NSCs markers^40^. The down-regulated DEGs mainly interacts with NSCs differentiation related genes and MNs differentiation related genes, in which GFAP was the marker of glial cells, Tubb3 was the marker of neurons, Sox10 could direct NSCs to oligodendrocyte lines^41^; Mecp2 was mainly expressed in neurons^42^; Shh, Ptc1, CXCR4, Nkx2-2, Olig1, Irx3, Crebbp, Tle1, Pou3f4, Foxg1, Ep300, Gdnf, Sv2a, Olig2, Slc18a3, Calca and Rbfox3 was related to motor neuron differentiation^43^. Above all, these genes interacting with DEGs were closely related to the proliferation and differentiation of NSCs, and most of these genes were also VTGs of DEmiRNAs. In addition, the further study also found that Dicer1, an important gene of miRNA biogenesis, and ago3, a related gene that played an inhibitory role, were the VTGs of miR-31-5p, suggesting that the change of miRNA expression profile in early stage of NSCs after miR-31 overexpressed might be related to this. At the same time, in addition to the fact that some DEGs were VTGs or PTGs of miR-31-5p, there were interaction networks between DEGs and some VTGs of miR-31. Among them, Stat3 was an important transcription factor regulating the expression of Gfap^44^; Sod2 played an important role in the regulation of cell cycle^45^; and Gsk3b could be used as a signal "node" to coordinate multiple key signaling pathways in NSCs^46,47^. Based on the above analysis, we conclude that the action mechanism of miR-31 in early stage of NSCs after overexpression was not only to inhibit its target gene, but also to expand its action scope by regulating the expression of other miRNAs, ultimately increasing the expression of genes or miRNAs related to NSCs stemness maintenance, and inhibiting NSCs, especially MNs, differentiation-related genes, through protein interaction and DEmiRNAs, to maintain the undifferentiated state of NSCs. All these indicated that miR-31 is a stemness maintenance gene of NSCs and has a negative regulatory role in the differentiation of NSCs into MNs. After interfering with the expression of miR-31 in spinal cord-derived NSCs, we further found that the expression levels of ChAT, Hb9, Nkx6.1, Nkx6.2, Isl1, Lhx3, and Olig2 genes increased in different degrees. At present, previous studies have shown that Olig2, Nkx6.1 and Nkx6.2 are downstream class II transcription factors of Shh. The mutual inhibition between Olig2, Nkx6.1, Nkx6.2 and Irx3, Dbx2, Dbx1 can ultimately determine the boundary between pMN and other domains in the spinal cord^43,48,49^. Hb9 (Mnx1)^50^, Isl1^51^ and ChAT^52^ are markers of MNs; Lhx3 can bind to Isl1 to form a complex, which plays an important role in the generation of specific motor neurons^53^. Meanwhile, when miR-31 was overexpressed in NSCs, the expression of these important MNs related genes decreased, while the expression of Nestin (a NSCs marker) increased, which was opposite to that when interfering with miR-31. These suggest that interference with miR-31 expression can induce NSCs to produce MNs like cells, while overexpression of miR-31 can maintain NSCs stemness. This further confirms our above conjecture about the role of miR-31 in NSCs from the above sequencing results. This study deepens our understanding of the role of miR-31 in NSCs, provides an effective candidate target for effectively inducing the differentiation of NSCs into MNs, and lays a foundation for us to effectively apply NSCs to the clinical treatment of motor neuron diseases.

## Methods

### Ethics statement

All animal procedures were performed according to guidelines developed by the China Council on Animal Care and protocols were approved by the Animal Care and Use Committee of Shanxi Province, China. The permit numbers are SCXK2009-0001.

### Sample preparation and experimental grouping

According to our previous study^13^, we cultured spinal cord-derived NSCs obtained from Balb/c mice embryos on days 16. Spinal cords were mechanically dissected using sterile technique under a dissecting microscope. Discarded the pia mater spinalis, and triturated spinal cord gently with a pipette to dissociate cells. Centrifuged dispersed cells at 337 × *g* for 5 min to get the cells’ pellet. Resuspended the pellet in the medium consisting of DMEM/F12, 2% B27 supplement with 20 ng/ml basic fibroblast growth factor (bFGF, R&D Systems) and 20 ng/ml epidermal growth factor (EGF, R&D Systems) at 37 ℃ in 5% CO_2_.

After cultured 14 days, NSCs were plated in 6-well culture plates, and then divided into two groups, one was miR-31 overexpression group which treated with miR-31 mimics (Thermo Fisher, MC10653); another group was miR-31 overexpression control group which treated with negative control of miRNA mimic (Thermo Fisher, 4464058). Each group contained six samples. The experimental process referred to the protocol of products.

### RNA extraction and Illumina sequencing

After 3 days of overexpression, the total RNA of miR-31 overexpression group and control group was extracted with Trizol reagent (Thermo Fisher), purified with anhydrous ethanol and treated with DNase. Randomly selected three samples in each group, mixed the total RNA of three samples in the same group, and obtained the final miR-31 overexpression group and its control group, which were finally used for sequencing analysis. Using Agilent Bioanalyzer 2100 (Agilent Technologies) to evaluate the integrity/quality of the two groups of total RNA.

Following the methods provided by TruSeq RNA Sample Preparation V2 Guideline (Illumina) and TruSeq Small RNA Sample Preparation Kit Version 2 (Illumina), constructed the mRNA and miRNA sequencing libraries, respectively. Sequencing analysis was performed respectively by using a HiSeq 2500 (Illumina) at Beijing Biomarker Technologies CO., LTD (Beijing, China).

### Analyses of RNA-Seq data and miRNA-Seq data

For the sequencing results of mRNA, FPKM (Fragments Per Kilobase of transcript per Million fragments mapped)^54^ was used as a measure of gene expression level, and EBSeq^55^ was used for differential expression analysis. For the results of miRNA sequencing, TPM algorithm^56^ was used to calculate the amount of miRNA expression in the samples, and IDEG6^57^ was used to analyze the differential expression. Both of them used Benjamini–Hochberg correction method to correct the significant p-value obtained from the original hypothesis test, and finally used False Discovery Rate (FDR) and Fold Change (FC), the ratio of expression between the two groups, as the key indicators for screening differentially expressed genes and miRNAs. Differentially expressed genes (DEGs) and differentially expressed miRNAs (DEmiRNAs) between the two samples were obtained using log2(FC) ≥ 1 and FDR ≤ 0.01 as screening criteria, respectively.

### q-PCR validation of DEGs and DEmiRNAs

Analyzed the relative expression levels of DEGs and DEmiRNAs in the remaining samples of the miR-31 overexpression group and its control group by real-time quantitative PCR. The primer information of the analyzed genes and miRNAs are listed in Supplementary Tables S1 and S2. The 2^−ΔΔCt^ method was used to analyze the relative gene expression level, and the Student's t-test was used to analyze the expression difference between the two groups. For both mRNAs and miRNAs, a p ≤ 0.05 after the Student’s t-test was considered statistically significant.

### Identification of PTGs and experimentally validated target genes (VTGs) of DEmiRNAs

The PTGs of DEmiRNAs were predicted by miRanda^58^ and RNAhybrid^59^, and the VTGs of DEmiRNAs were identified by DIANA-TarBase v.8^60^ and miRWalk^61^.

### Functional annotation and enrichment analysis

The DEGs and PTGs were compared with NR^62^, Swiss-Prot^63^, COG^64^, GO^65,66^, KEGG^67–69^ databases to obtain annotation information of target genes, and cluster analysis was performed.

### miRNA-mRNA regulatory network and protein–protein interactions (PPI) network

The miRNA-mRNA regulatory network is mainly constructed based on the opposite expression patterns of DEmiRNAs and DEGs, and PPI between DEGs and VTGs are constructed using STRING database (https://string-db.org/).

### Effects of overexpression or interference with miR-31 on the expression of NSCs and MNs related genes

NSCs were divided into four groups: interfering miR-31 group, interfering control group, overexpressing miR-31 group and overexpressing control group. mmu-miR-31-5p mimic, mmu-miR-31-5p mimics Negative Control, mmu-miR-31-5p inhibitor and mmu-miR-31-5p inhibitor Negative Control (Supplementary Table S6) were transfected by Lipofectamine RNAiMAX (Thermo Fisher) according to group. After incubation at 37 ℃ and 5% CO_2_ for 7 days, the expression of Nestin, ChAT, Hb9, Nkx6.1, Nkx6.2, Isl1, Lhx3 and Olig2 in each group was detected by q-PCR (the information of primers is listed in Supplementary Table S7).

## Supplementary information

Supplementary Tables.

## Data Availability

The datasets generated during and/or analyzed during the current study are available by request.
