# Supplementary material for: mRNA and miRNA expression profile reveals the role of miR-31 overexpression in neural stem cell
Source: Sci Rep. 2020 Oct 16;10:17537. doi: 10.1038/s41598-020-74541-8 (PMC7568549; doi:10.1038/s41598-020-74541-8)
Supplement: Supplementary file 1 — Supplementary Tables. [file 41598_2020_74541_MOESM1_ESM.pdf]

mRNA and miRNA Expression Profile Reveals the Role of miR-31 Overexpression in Neural Stem Cell

Pengfei Li[1,3], Yuantao Gao[2], Xiao Li[3], Feng Tian[3], Fei Wang[3], Yali Wang[3], Bichun Zhao[3], Ruxin Zhang[3], Chunfang Wang[3*]

1. Translational Medicine Research Center, Shanxi Medical University, Xinjian South Road 56, Taiyuan, 030001, Shanxi, People's Republic of China. sxmulpf@sxmu.edu.cn

2. Nanchang University, Nanchang 330000, China

3. Laboratory Animal Center, Shanxi Medical University, Xinjian South Road 56, Shanxi 030001, People's Republic of China

* Corresponding author. wangchunfang@sxmu.edu.cn

Table S1 Specific Forward and Reverse Oligonucleotide Primers Used for q-PCR Validation

Gene	Primer	
Ecel1	Forward Primer	CTGGCGCTCAAATACCTGGG
	Reverse Primer	GCGAACGAGTAGAAGTCCTGG
Pkd2l1	Forward Primer	TACAGCGACCCTCCTTCCC
	Reverse Primer	CCTCTGATGCTCCGACAGATATG
Syt17	Forward Primer	GTCAGAGGTGCTATGAGTCCA
	Reverse Primer	GGGGTCAAAGGAACATCGCT
Bsn	Forward Primer	GGAACAGCTTGACAGTGCAG
	Reverse Primer	GGACGCCGTTTCTGGTCTT
Mag	Forward Primer	CTGCCGCTGTTTTGGATAATGA
	Reverse Primer	CATCGGGGAAGTCGAAACGG
En2	Forward Primer	ACTGCACGCGCTATTCTG
	Reverse Primer	ACCTGTTGGTCTGAAACTCAG
Actb	Forward Primer	AATCGTGCGTGACATCAAAG
	Reverse Primer	AAGGAAGGCTGGAAAAGAGC

Table S2 The MicroRNA Assays Used for q-PCR Validation

miRNA	Assay ID (Thermo Fisher)
mmu-miR-31-5p	000185
mmu-miR-106b-5p	000442
mmu-miR-130a-3p	000454
mmu-miR-135a-5p	000460
mmu-let-7d-5p	002283
mmu-let-7a-5p	000377
mmu-miR-221-5p	464489_mat
U6	001973

Table S3 The Differentially Expressed mRNAs in the miR-31 Overexpression Group Compared with Control Group

ID	gene symbol	FDR	log2FC	regulated
ENSMUSG00000001864	Aif1l	6.11E-15	1.111771525	up
ENSMUSG00000026247	Ecel1	1.39E-05	1.122647955	up
ENSMUSG00000037578	Pkd2l1	0.000185611	1.087336415	up
ENSMUSG00000044083	Efcab8	1.09E-05	1.470037817	up
ENSMUSG00000050195	Scd4	0.000149391	1.372345899	up
ENSMUSG00000058420	Syt17	0.005379485	1.470153928	up
ENSMUSG00000064220	Hist2h2aa2	0.000143465	1.015684527	up
ENSMUSG00000066878	Gm10184	0.000514424	4.40662989	up
Mouse_newGene_1157		0.001273748	1.094317873	up
Mouse_newGene_1823		0.005765096	1.021452923	up
Mouse_newGene_1830		0	2.095696295	up
Mouse_newGene_2228		0	6.495983747	up
Mouse_newGene_861		1.39E-12	1.424551558	up
ENSMUSG00000009487	Otog	0.000155254	-2.138862034	down
ENSMUSG00000020010	Vnn3	0.000259711	-2.493341786	down
ENSMUSG00000022491	Glycam1	7.29E-12	-1.00124495	down
ENSMUSG00000030222	Rerg	2.91E-05	-1.173646218	down
ENSMUSG00000031450	Grk1	0.002058483	-1.192255735	down
ENSMUSG00000031906	Smpd3	0	-1.768817434	down
ENSMUSG00000032589	Bsn	8.44E-06	-1.362676829	down
ENSMUSG00000036634	Mag	0	-1.116324017	down
ENSMUSG00000037754	Ppp1r16b	0	-1.239808731	down
ENSMUSG00000037996	Slc24a2	0	-1.269237924	down
ENSMUSG00000039095	En2	0.00449843	-1.048815128	down
ENSMUSG00000046623	Gjb4	0.009854119	-3.089812219	down
ENSMUSG00000047109	Cldn14	0.006577204	-1.418900597	down
ENSMUSG00000051000	Fam160a1	0.001033936	-1.031188064	down
ENSMUSG00000052135	Foxo6	0.008535573	-1.066454691	down
ENSMUSG00000055415	Atp10b	2.66E-05	-2.12099228	down
ENSMUSG00000067276	Capn6	4.48E-12	-1.010036871	down
ENSMUSG00000079507	H2-Q1	0	-6.57186422	down
Mouse_newGene_1145		1.90E-05	-1.353731753	down
Mouse_newGene_1852		0.001886957	-1.033415344	down
Mouse_newGene_1858		0.007856949	-4.154562916	down
Mouse_newGene_362		0.000488433	-1.745726762	down

Table S4 The Differentially Expressed miRNAs in the miR-31 Overexpression Group Compared with Control Group

ID	FDR	log2FC	regulated
unconservative_4_517095	0	29.67329677	up
unconservative_17_331075	0	27.81433999	up
unconservative_10_85637	0	25.78576375	up
mmu-miR-669b-3p	1.31E-05	25.04880646	up
mmu-miR-20b-5p	0.001469277	24.31181316	up
unconservative_8_722210	0.001469277	24.31181316	up
unconservative_12_166966	0.003101294	24.1418795	up
mmu-miR-31-5p	0	5.481680359	up
mmu-miR-466g	5.39E-05	4.305769463	up
mmu-miR-491-5p	0	3.890727634	up
unconservative_10_77518	0.001917784	3.765183369	up
mmu-miR-6418-3p	0.003701579	3.627674177	up
mmu-let-7g-3p	0	3.528156317	up
mmu-miR-466i-3p	0.006981324	3.475664156	up
mmu-miR-466q	0.006981324	3.475664156	up
mmu-miR-130b-3p	0	3.305769463	up
mmu-miR-8114	0.00026575	3.305769463	up
mmu-miR-350-3p	0	3.240267762	up
mmu-miR-135a-5p	0	3.198250805	up
unconservative_5_596467	0	3.09584029	up
unconservative_5_596468	0	3.09584029	up
mmu-miR-101b-3p	0	3.091401245	up
mmu-miR-376b-3p	8.86E-06	2.949614741	up
mmu-miR-210-5p	0	2.946402702	up
mmu-miR-877-5p	0	2.930257168	up
mmu-miR-3102-3p	0	2.883424987	up
mmu-miR-8112	3.37E-05	2.82931617	up
mmu-miR-324-5p	0	2.816713371	up
mmu-miR-297a-3p	0	2.750548738	up
mmu-miR-297b-3p	0	2.750548738	up
mmu-miR-297c-3p	0	2.750548738	up
mmu-miR-17-3p	0	2.728977591	up
mmu-miR-154-5p	0	2.720806962	up
mmu-miR-5113	0.000877637	2.720806962	up
mmu-miR-136-5p	0	2.705699659	up
unconservative_7_654738	0.000123127	2.698096163	up
mmu-miR-133b-3p	0.001609264	2.62769496	up
mmu-miR-193b-3p	0	2.559999489	up
mmu-miR-574-3p	0	2.491728727	up
mmu-miR-500-3p	0	2.491633867	up
mmu-miR-378b	0	2.470825549	up

mmu-miR-135b-5p	0.004965089	2.421237687	up
unconservative_14_230657	0.004965089	2.421237687	up
mmu-miR-190a-5p	0	2.405302538	up
unconservative_12_150502	0.000343677	2.376168068	up
mmu-miR-299a-5p	0	2.331299769	up
mmu-miR-671-5p	0	2.305752144	up
mmu-miR-466d-3p	1.72E-05	2.259964516	up
mmu-miR-18a-5p	0	2.244345816	up
mmu-miR-324-3p	0	2.2386497	up
mmu-miR-132-3p	0	2.219886625	up
mmu-miR-196b-3p	0.003876226	2.21265746	up
mmu-miR-34c-3p	0	2.182270885	up
mmu-miR-30b-5p	0	2.161642536	up
unconservative_X_804847	0.000218259	2.142259819	up
mmu-miR-495-3p	0	2.100179361	up
mmu-miR-345-5p	0	2.068503248	up
mmu-miR-425-5p	0	2.004585048	up
mmu-miR-187-3p	0	1.96567804	up
mmu-miR-365-3p	0	1.961164457	up
mmu-miR-188-5p	0	1.943193816	up
mmu-miR-547-3p	0	1.922439129	up
mmu-miR-151-5p	0	1.905735172	up
mmu-miR-532-3p	0	1.898982506	up
mmu-miR-186-3p	0.003727358	1.890736293	up
mmu-miR-214-3p	0	1.890717798	up
mmu-miR-374b-5p	0	1.875635802	up
mmu-miR-669a-3p	0	1.863741303	up
mmu-miR-669o-3p	0	1.863741303	up
mmu-miR-467d-3p	0	1.844927017	up
mmu-miR-467a-3p	0	1.829329724	up
mmu-miR-376b-5p	0.002809863	1.820350429	up
mmu-miR-1249-3p	0	1.807108675	up
mmu-miR-664-3p	0.000207713	1.800532897	up
mmu-miR-19b-3p	0	1.783677987	up
mmu-miR-877-3p	4.53E-06	1.77970221	up
mmu-miR-29a-5p	4.53E-06	1.772894644	up
mmu-miR-29b-3p	0	1.768262163	up
unconservative_7_653856	0	1.765194705	up
mmu-miR-212-3p	0	1.728977591	up
mmu-miR-21a-3p	0	1.72077906	up
mmu-miR-3093-3p	0	1.72077906	up
mmu-miR-16-5p	0	1.717503892	up
mmu-miR-326-3p	0	1.680651009	up
mmu-miR-3099-3p	0	1.675002015	up

mmu-miR-153-3p	0	1.661913273	up
mmu-miR-376c-3p	0.002456392	1.659391169	up
mmu-miR-378a-5p	0	1.656661913	up
mmu-miR-467d-5p	0.000856518	1.653687199	up
mmu-miR-106b-5p	0	1.650944764	up
mmu-miR-700-5p	0	1.648897781	up
mmu-miR-129-1-3p	0	1.645386433	up
mmu-miR-29c-5p	0	1.638337925	up
mmu-miR-129-2-3p	0	1.610886515	up
mmu-miR-126a-5p	0.000465919	1.605329744	up
mmu-miR-365-1-5p	0.000465919	1.605329744	up
mmu-miR-15a-5p	0	1.605310053	up
mmu-miR-196b-5p	0	1.600506349	up
mmu-miR-124-3p	0.000346371	1.585879674	up
mmu-miR-34b-3p	0	1.573617887	up
mmu-miR-466b-3p	0	1.568799539	up
mmu-miR-466c-3p	0	1.568799539	up
mmu-miR-466p-3p	0	1.568799539	up
mmu-miR-574-5p	0	1.568785857	up
mmu-miR-708-5p	0	1.567925669	up
mmu-miR-190b-5p	0.005710763	1.528171162	up
mmu-miR-670-3p	0.000215034	1.503699848	up
mmu-miR-223-3p	0	1.502786684	up
mmu-miR-31-3p	0	1.496896114	up
mmu-miR-466a-3p	0	1.495590285	up
mmu-miR-466e-3p	0	1.495590285	up
mmu-miR-652-3p	0	1.490760106	up
mmu-miR-669l-5p	0.003133053	1.490183123	up
mmu-miR-342-3p	0	1.487810689	up
mmu-miR-467c-5p	4.53E-06	1.475693845	up
mmu-miR-219a-5p	0.002324186	1.475687248	up
mmu-miR-486a-3p	0.000919759	1.443274167	up
mmu-miR-152-3p	0	1.436588049	up
mmu-miR-3081-3p	0	1.423452668	up
mmu-miR-138-1-3p	3.77E-05	1.421237687	up
mmu-miR-130a-5p	0.003455616	1.421237687	up
mmu-miR-34a-5p	0	1.400100971	up
mmu-miR-193a-3p	0.002075034	1.35307392	up
mmu-miR-322-5p	0	1.343370137	up
mmu-miR-32-5p	0	1.340906224	up
mmu-miR-486a-5p	0	1.312775207	up
mmu-miR-760-3p	0.003023566	1.305766865	up
mmu-miR-1839-3p	0.004019152	1.305763896	up
mmu-miR-133a-3p	0	1.305752144	up

mmu-let-7i-3p	0	1.300517536	up
mmu-miR-29c-3p	0	1.297247282	up
mmu-miR-935	0	1.277180523	up
mmu-miR-32-3p	0.003227338	1.259964516	up
mmu-miR-138-5p	0	1.253859223	up
mmu-miR-139-5p	0	1.249704808	up
mmu-miR-669a-5p	0	1.249173456	up
mmu-miR-669p-5p	0	1.249173456	up
mmu-miR-17-5p	0	1.237681362	up
mmu-miR-134-5p	0	1.224144073	up
mmu-miR-379-5p	0	1.223105345	up
mmu-miR-204-5p	0	1.216971976	up
mmu-miR-30e-5p	0	1.212969701	up
mmu-miR-28a-5p	0	1.171972333	up
mmu-miR-503-5p	0	1.171938245	up
mmu-miR-700-3p	0	1.168250363	up
mmu-miR-149-5p	0	1.161365584	up
mmu-miR-1191a	1.72E-05	1.161359153	up
mmu-miR-142a-3p	0	1.161253439	up
mmu-miR-140-5p	0	1.154452131	up
mmu-miR-130a-3p	0	1.152400999	up
mmu-miR-3473e	0.003783733	1.145307083	up
mmu-miR-206-3p	8.86E-06	1.133983355	up
unconservative_8_723625	0.002248932	1.13173107	up
mmu-miR-411-5p	0	1.129375066	up
mmu-miR-20a-5p	0	1.121571328	up
mmu-miR-151-3p	0	1.118327543	up
mmu-miR-582-5p	1.31E-05	1.113102762	up
unconservative_12_147376	4.53E-06	1.094250935	up
unconservative_7_691663	4.53E-06	1.094250935	up
mmu-miR-155-5p	0	1.091401416	up
mmu-miR-218-5p	0	1.080196225	up
mmu-miR-24-3p	0	1.037565791	up
mmu-miR-126a-3p	0	1.029616621	up
mmu-miR-335-5p	0	1.028221645	up
unconservative_12_178860	4.53E-06	1.024854959	up
mmu-miR-345-3p	0	1.020915666	up
mmu-miR-142a-5p	0	1.018727464	up
mmu-miR-196a-5p	0	1.016248466	up
mmu-miR-193a-5p	0.007548246	1.000917173	up
mmu-miR-221-5p	0	-1.004027716	down
mmu-miR-340-3p	0	-1.071765263	down
mmu-miR-450a-1-3p	0.003815897	-1.078906455	down
mmu-miR-1843a-3p	0.005715265	-1.086566309	down

mmu-miR-598-3p	0	-1.098875255	down
mmu-miR-224-5p	0.000848097	-1.159898119	down
mmu-miR-130b-5p	0.000430732	-1.170695871	down
mmu-let-7a-1-3p	0	-1.20373481	down
mmu-let-7c-2-3p	0	-1.20373481	down
mmu-let-7j	0	-1.243803652	down
mmu-miR-541-5p	0	-1.261488484	down
mmu-miR-1964-3p	5.39E-05	-1.320441804	down
mmu-let-7g-5p	0	-1.385503098	down
mmu-miR-212-5p	0	-1.458429023	down
mmu-let-7f-5p	0	-1.498827373	down
mmu-let-7c-1-3p	0	-1.544104663	down
mmu-miR-543-3p	0.002867628	-1.809668788	down
mmu-miR-6516-5p	0.002765933	-1.957225976	down
mmu-let-7a-5p	0	-1.989481256	down
mmu-let-7d-5p	0	-2.218560066	down

Table S5 The Opposite Expression Patterns of DEGs and DEmiRNAs

Regulation of DEmiRNAs	miRNA	Target Gene	Regulation of DEGs
up	mmu-miR-124-3p	Smpd3	down
up	mmu-miR-124-3p	Bsn	down
up	mmu-miR-124-3p	Slc24a2	down
up	mmu-miR-124-3p	En2	down
up	mmu-miR-124-3p	Gjb4	down
up	mmu-miR-124-3p	Fam160a1	down
up	mmu-miR-124-3p	Foxo6	down
up	mmu-miR-124-3p	Capn6	down
up	mmu-miR-155-5p	Otog	down
up	mmu-miR-155-5p	Vnn3	down
up	mmu-miR-155-5p	Smpd3	down
up	mmu-miR-155-5p	Bsn	down
up	mmu-miR-155-5p	Mag	down
up	mmu-miR-155-5p	Gjb4	down
up	mmu-miR-155-5p	Fam160a1	down
up	mmu-miR-155-5p	Foxo6	down
up	mmu-miR-16-5p	Smpd3	down
up	mmu-miR-16-5p	Bsn	down
up	mmu-miR-16-5p	En2	down
up	mmu-miR-223-3p	Bsn	down
up	mmu-miR-223-3p	Mag	down
down	mmu-let-7g-5p	Hist2h2aa2	up
up	mmu-miR-133a-3p	Otog	down
up	mmu-miR-133b-3p	Otog	down
up	mmu-miR-138-5p	Smpd3	down
up	mmu-miR-138-5p	Bsn	down
up	mmu-miR-153-3p	Bsn	down
up	mmu-miR-153-3p	En2	down
up	mmu-miR-188-5p	Capn6	down
up	mmu-miR-193b-3p	Bsn	down
up	mmu-miR-193b-3p	Slc24a2	down
up	mmu-miR-19b-3p	Slc24a2	down
up	mmu-miR-19b-3p	Fam160a1	down
up	mmu-miR-31-5p	Fam160a1	down
up	mmu-miR-322-5p	Otog	down
up	mmu-miR-322-5p	Vnn3	down
up	mmu-miR-466i-3p	Otog	down
up	mmu-miR-466i-3p	Bsn	down
up	mmu-miR-708-5p	Bsn	down
up	mmu-miR-708-5p	En2	down
up	mmu-miR-101b-3p	Bsn	down
up	mmu-miR-17-5p	Slc24a2	down

up	mmu-miR-206-3p	Bsn	down
up	mmu-miR-218-5p	Bsn	down
up	mmu-miR-24-3p	Bsn	down
up	mmu-miR-28a-5p	Fam160a1	down
up	mmu-miR-297b-3p	Bsn	down
up	mmu-miR-29a-5p	Capn6	down
up	mmu-miR-29b-3p	Capn6	down
up	mmu-miR-30e-5p	En2	down
up	mmu-miR-31-3p	Bsn	down
up	mmu-miR-324-3p	Slc24a2	down
up	mmu-miR-342-3p	Bsn	down
up	mmu-miR-374b-5p	En2	down
up	mmu-miR-378a-5p	Capn6	down
up	mmu-miR-425-5p	Slc24a2	down
up	mmu-miR-466a-3p	Bsn	down
up	mmu-miR-466b-3p	Bsn	down
up	mmu-miR-466c-3p	Bsn	down
up	mmu-miR-466d-3p	Bsn	down
up	mmu-miR-466e-3p	Bsn	down
up	mmu-miR-466p-3p	Bsn	down
up	mmu-miR-466q	Fam160a1	down
up	mmu-miR-467c-5p	Smpd3	down
up	mmu-miR-486a-5p	Otog	down
up	mmu-miR-500-3p	Foxo6	down
up	mmu-miR-532-3p	Capn6	down

Table S6 mirVana mimics & inhibitors of mmu-miR-31-5p

mirVana mimics & inhibitors	Cat# (Thermo Fisher)
mirVana miRNA mimics	4464066
mirVana miRNA mimics Negative Control	4464058
mirVana miRNA inhibitor	4464084
mirVana miRNA inhibitor Negative Control	000460

Table S7 Specific Forward and Reverse Oligonucleotide Primers of NSCs and MNs Related Genes Used for q-PCR

Gene	Primer	
Nestin	Forward Primer	CCCTGAAGTCGAGGAGCTG
	Reverse Primer	CTGCTGCACCTCTAAGCGA
ChAT	Forward Primer	CCATTGTGAAGCGGTTTGGG
	Reverse Primer	GCCAGGCGGTTGTTTAGATACA
Hb9	Forward Primer	GAACACCAGTTCAAGCTCAACA
	Reverse Primer	GCTGCGTTTCCATTTCATTCG
Nkx6.1	Forward Primer	CTGCACAGTATGGCCGAGATG
	Reverse Primer	CCGGGTTATGTGAGCCCAA
Nkx6.2	Forward Primer	GCATGACCGAGAGCCAAGT
	Reverse Primer	GCATCCGAGTCTTGCTTCTTTTT
Isl1	Forward Primer	ATGATGGTGGTTTACAGGCTAAC
	Reverse Primer	TCGATGCTACTTCACTGCCAG
Lhx3	Forward Primer	AGGCGGACTACGAAACAGC
	Reverse Primer	CGAAGTGTTGTAGGCGCTCT
Olig2	Forward Primer	TCCCCAGAACCCGATGATCTT
	Reverse Primer	CGTGGACGAGGACACAGTC
Actb	Forward Primer	GGCTGTATTCCCCTCCATCG
	Reverse Primer	CCAGTTGGTAACAATGCCATGT
